# Validity of EQ-5D utility index and minimal clinically important difference estimation among patients with chronic obstructive pulmonary disease

**DOI:** 10.1186/s12890-020-1116-z

**Published:** 2020-03-23

**Authors:** Eunmi Bae, Sang-Eun Choi, Haeyoung Lee, Gyeongseon Shin, Daewon Kang

**Affiliations:** 1 College of Pharmacy, Korea University, 2511 Sejong-ro, Sejong, 30019 South Korea; 2 University of Maryland School of Pharmacy, Baltimore, MD USA

**Keywords:** Chronic obstructive pulmonary disease, Health-related quality of life, Utility, EQ-5D, MCID, Korea

## Abstract

**Background:**

The discriminatory ability of multi-attribute utility (MAU) measures compared to condition-specific measures (CSM) in assessing health-related quality of life (HRQoL) among patients with chronic obstructive pulmonary disease (COPD) is an unsettled issue. This study investigated the quality of life of patients with COPD with three different HRQoL instruments and examined whether they could differentiate between adjacent severity groups in a statistically and clinically meaningful manner. In the process, the minimal clinically important differences (MCID) of the EQ-5D utility index were estimated.

**Methods:**

Cross-sectional survey data were collected from patients with mild to very severe COPD in South Korea. In addition to demographic and clinical information, the following HRQoL questionnaires were used: The three-level five-dimensional Euro-Quality of Life tool (EQ-5D-3L), the EQ-Visual Analog Scale (EQ-VAS), and the Chronic Obstructive Pulmonary Disease Assessment Test (CAT). Patients’ health-related quality of life was analyzed with reference to severity groups based on the Global Initiative for Chronic Obstructive Lung Disease (GOLD) classification. To investigate the discriminatory ability of the HRQoL instruments between COPD severity groups, tests examining variance, covariance, and standardized mean difference were performed. After estimating the MCID of the EQ-5D utility index using the anchor-based method, we investigated whether the differences in the EQ-5D utility scores between groups exceeded the clinically meaningful minimum level.

**Results:**

A total of 298 patients completed this study. All the quality of life scores showed statistically significant differences between the GOLD severity groups. The pooled MCID estimate for the EQ-5D utility index was 0.028 (range: 0.017–0.033). Even after adjusting for other factors affecting quality of life, the EQ-5D utility index differentiated the GOLD groups well.

**Conclusions:**

We conclude that the EQ-5D utility index is a valid instrument for measuring the quality of life of patients with COPD, and the pooled MCID estimate for the EQ-5D utility index was 0.028.

## Background

Chronic obstructive pulmonary disease (COPD) is characterized by airflow limitation, and it affects the quality of life of patients owing to various symptoms, acute exacerbation, and complications. There is no cure for COPD, therefore, the purpose of pharmacological and nonpharmacologic treatments is to mitigate symptoms and improve quality of life [1, 2]. Therefore, the health-related quality of life (HRQoL) of patients with COPD has been considered an important disease outcome in recent clinical studies [3, 4].

Tools assessing HRQoL should be able to differentiate it according to the patient’s clinical status [5]. The progression of COPD in the utility-based decision analytic model is mainly based on discrete clinical stages or continuous changes in pulmonary function [6, 7]. As a representative staging system, the Global Initiative for Chronic Obstructive Lung Disease (GOLD) classification uses spirometry airflow limitations measured with the forced expiratory volume per second (FEV_1_). FEV_1_ is the most useful predictor of clinical outcomes such as mortality and hospitalization rates, and it provides important information on the clinical status of patients at the population level. However, its association with symptoms or patients’ quality of life is somewhat weak [8, 9]. Pickard et al. [10] and Einarson et al. [11] reported that the utility measure reflects the clinical severity of COPD well, as evidenced by lower utility scores when disease severity is higher. However, many other studies have reported that the discriminatory ability of a multi-attribute utility (MAU) measure, such as the five-dimensional EuroQol (EQ-5D) utility index, is limited with respect to measuring the quality of life of patients with COPD [4, 10–14]. Therefore, there is an increasing tendency to use condition-specific measures (CSMs), which are more sensitive to the detection of small treatment effects. CSMs that are designed to capture the clinical consequences or small changes in a certain disease are preferred for clinical studies. However, as such measures are not preference based, their use in economic evaluation is extremely limited [15]. Among the existing preference-based utility measures, the EQ-5D utility index is the most preferred tool based on the multi-attribute utility theory (MAUT) because its valuation studies have been conducted in many countries. It is necessary to examine the discrimination ability of the EQ-5D utility index in evaluating the quality of life of patients with COPD. This differentiation is not only important in the statistical sense but also with respect to the clinical meaning of the results. Recently, as Patient Reported Outcomes (PROs) have come to play an important role in the evaluation of treatments or interventions, interest in minimal clinically important differences (MCID), which facilitate the interpretation of PRO scores, has been growing. However, though MCID has been studied with reference to several disease areas, there is no consensus on the MCID of the EQ-5D utility index as yet [16].

This study aims to investigate whether the EQ-5D utility index is a valid tool for assessing the quality of life of patients with COPD. To this end, we examined the quality of life of patients with COPD using data from a cross-sectional patient survey. Additionally, we estimated the MCID of the EQ-5D utility index using various established methods. We then tried to determine whether the EQ-5D utility index usefully differentiates statistically and clinically between the severity groups.

## Methods

### Study subjects

A multicenter, non-interventional, cross-sectional study was conducted from August to December 2014, at the pulmonary division of three educational hospitals in Seoul, South Korea. The study protocol was approved by the Institutional Review Board (IRB) at each hospital (approval numbers: ED14144; KUGH14146; KC14QIMI0470) and all patients were informed about the study and consented to participate by signing the form recommended by the IRBs. Study subjects had mild to very severe COPD diagnosed before 1 January 2013. The inclusion criteria were age over 40 years, FEV_1_/forced vital capacity (FVC) ratio of less than 70% after bronchodilator administration, and previous or current smoking history of 10 or more pack-years [1, 12]. The exclusion criteria were exacerbation cases occurring within 6 weeks or cardiovascular events within 3 months, or participation in other clinical trials since January 2013. By excluding the effects of acute events, it is possible to pursue clinical stability of the patient, which is beneficial for measuring the quality of life.

### Disease severity classification

Patients were classified according to the GOLD severity grade, which is based on post-bronchodilator FEV_1_% predicted. The definition of GOLD stages is as follows: GOLD 1 (mild) as FEV_1_% predicted ≥80%; GOLD 2 (moderate) as FEV_1_% predicted ≥50% to less than 80%; GOLD 3 (severe) as FEV_1_% predicted ≥30% to less than 50%; and GOLD 4 (very severe) as FEV_1_% predicted less than 30%.

### Clinical data

Demographic information was collected, including each patient’s age, gender, BMI, smoking history, and socioeconomic status. Additionally, clinical information was gathered, including the duration of diagnosis, pulmonary function, comorbidities (including angina, myocardial infarction (MI), congestive heart failure (CHF), atrial fibrillation (AF), hypertension, diabetes, metabolic syndrome, gastroesophageal reflux disease (GERD), osteoporosis, anxiety or depression, lung cancer, asthma, arthritis, and anemia), history of tuberculosis, and prescription records. Information on resource utilization for 1 year was also collected. All these data were collected from recent medical records at the time of the survey.

COPD exacerbation is generally defined as a sustained worsening of the patient’s condition beyond normal day-to-day variations, that is acute in onset and necessitates a change from the usual medication [17, 18]. For the present study, we operationally defined COPD exacerbations as cases in which the patient was prescribed oral corticosteroids and antibiotics simultaneously. Additionally, hospital admission status was also investigated.

### QoL data

Our survey instruments were the three-level five-dimensional EuroQol (EQ-5D-3L), EQ-Visual Analog Scale (EQ-VAS) [19], and Chronic Obstructive Pulmonary Disease Assessment Test (CAT) [20]. All questionnaires were self-administered under the supervision of the clinician.

#### EuroQol

The EQ-5D has been used widely in a variety of clinical areas and countries to evaluate health-related quality of life. This questionnaire consists of a descriptive section defining health status (EQ-5D-3L) and a single index value that captures a self-rating of health status on a Visual Analog Scale (EQ-VAS). The descriptive section includes the five dimensions of mobility, self-care, usual activities, pain/discomfort, and anxiety/depression. Each dimension is divided into the following three levels: No problem, some or moderate problems, and extreme problems. The resulting health state can be defined by a 5-digit number that combines the levels from each of the five dimensions (e.g., 11231). The EQ-VAS ratings are a quantitative measure ranging from 100 (representing the best imaginable health state) to 0 (representing the worst imaginable health state) [19]. The information derived from the EQ-5D self-classifier can also be converted into a single summary index (the EQ-5D utility index) by applying scores from a national valuation set generated from a population-based preference survey. The value set used in the present study was derived from a representative South Korean population sample with 1307 subjects using the time trade-off (TTO) method [21]. A total of 101 health states were directly valued and used to develop the model for all 243 health states defined by the EQ-5D-3L. As in many other countries, South Korean health authorities recommend the EQ-5D for cost-effective analyses.

#### CAT

The CAT has been developed for understanding and grading the impact of COPD on health-related quality of life [22]. It is short, simple, and easy to self-administer, and it is designed for routine use in clinical practice. It comprises eight items covering cough, phlegm, chest tightness, breathlessness, activity limitation, confidence, sleeping, and energy, and each item is scored from 0 to 5 points. The total score ranges from 0 to 40, with higher scores indicating worse quality of life. The linguistic validity and reliability of the South Korean version of the questionnaire used in this study have been verified [23, 24]. The recent GOLD guidelines recommend the use of the Modified Medical Research Council (mMRC) or CAT for symptoms assessment in patients with COPD.

### Data analysis

#### Description of sample

Participants’ characteristics were grouped by GOLD severity and summarized using mean and standard deviation (SD) for continuous variables, and frequencies for categorical variables. Analysis of variance (ANOVA) and chi-square tests were then performed to test the differences between groups.

#### Investigation of validity

We examined the validity of the EQ-5D utility index both statistically and clinically. The cross-sectional construct validity of the instruments in terms of their ability to differentiate different health states was tested using known severity group. Also, all the utility differences between groups were compared to MCID to determine if they are clinically meaningful. For construct validity, we used post-hoc analysis to determine whether there was a significant difference in quality of life scores between neighboring groups in addition to ANOVA. To investigate the sensitivity of discriminatory ability between the health status of patients with COPD from different groups, the effect size using Cohen’s *d* (based on the standardized mean difference between two populations) was calculated [25]. Large effect sizes indicate that there is no problem in detecting a consistent difference between groups based on their means. Owing to the skewed nature of the EQ-5D utility index data, non-parametric/parametric correlation coefficients (Spearman’s rank/Pearson correlation coefficient) were calculated to assess the association between the HRQoL scores and lung function. Analysis of covariance (ANCOVA) was used to compare the quality of life of each group after adjusting for factors that might affect the quality of life of patients with COPD, and the Least Significant Difference (LSD) was used for post-hoc analysis. Background characteristics such as age, gender, smoking years, insurance type, employment status, and number of comorbidities were used as control variables in these analyses. Although the assumptions of normality and constant variance were not met in the EQ-5D utility index data, the distributions of EQ-VAS and CAT scores did not deviate significantly from normal distributions. Therefore, we decided to use parametric analyses and applied ANOVA/ANCOVA. Additionally, the Kruskal-Wallis test and the subsequent Wilcoxon rank-sum test to compare two specific groups were conducted; the results of these non-parametric analyses are presented in the supplementary Table (S1). All statistical analyses were conducted using IBM SPSS 22 and STATA v.13.

#### MCID estimation

The MCID was estimated to determine where utilities by severity group reflect clinically meaningful differences. MCID was first used by Jaeschke et al. in 1989 to identify the smallest change that is important to patients. It can be estimated using distribution-based and anchor-based methods [26–30].

##### Distribution-based method

The distribution-based method uses the variation measured in the PRO score. In this study, we used the following three assumptions.

**(1) 1/2 SD approach:** It was assumed that 0.5*SD would correspond to the MCID. The SD is the variation among individual scores, and approximately half an SD appeared to be the limit of discriminability of changes based on a psychological theory, which has been empirically derived in many health-related quality of life studies of chronic disease and thus does not constitute an arbitrary statistical threshold [26, 31]; (**2) SEM approach:** After estimating measurement error using the following formula: $\mathrm{standard}\ \mathrm{error}\ \mathrm{of}\ \mathrm{the}\ \mathrm{measurement}\ \left(\mathrm{SEM}\right)=\mathrm{SD}\times \sqrt{1-\mathrm{reliability}\ \mathrm{of}\ \mathrm{a}\ \mathrm{measure}}$, we assumed that SEM would correspond to the MCID. Test-retest reliability refers to the consistency of a measurement, and it can be assessed with the Intraclass Correlation Coefficient (ICC), which is frequently reported as a common metric in reliability studies [32]. The ICC was obtained from a validity study of the EQ-5D-3L involving a representative sample of the South Korean population (0.61) [33]. Where the reliability of a measure is less than 0.75, 1 SEM may be a more stringent criterion than 0.5 SD [31]; (**3) Cohen’s approach:** Using Cohen’s formulation, which is generally accepted as a benchmark, we assumed that the SD of the utility score multiplied by 0.2, corresponding to a small effect size, would correspond to the MCID [27].

##### Anchor-based method

The anchor-based approach is a method of comparing changes in the PRO score using an anchor or external criterion. The external criterion should have a proven association with the PROs and a minimum correlation of 0.30–0.35 is recommended [28]. We considered the statistical correlation and clinical relevance of the EQ-5D utility index, and clinical indicators such as the FEV_1_% predicted, EQ-VAS, and CAT scores were selected as anchors. The FEV_1_ is the most common clinical indicator of COPD prognosis in terms of repeatability. However, it is reasonable to use the MCID for the FEV_1_% predicted value instead of the FEV_1_ alone because this was not a longitudinal study involving data on changes in pulmonary function values for individual patients. Although no MCID study has utilized the FEV_1_% predicted, a 5–10% difference from the baseline is considered clinically significant and a difference of less than 3% clinically insignificant [34, 35]. Therefore, we used 5–10% differences in the FEV_1_% predicted as an anchor. The MCID of the EQ-VAS and CAT scores for patients with COPD were estimated to be 8 [36] and between 2 and 3 in most previous studies [37–41], respectively. Additionally, we constructed a simple regression model in which the external criteria used as an anchor were the independent variables and the utility was the dependent variable. The MCID of the utility index was estimated by multiplying the known MCID of the external criterion by the coefficient of the regression model. The relevance of each anchor to the EQ-5D utility index was computed as Spearman’s rank correlation coefficient.

The MCID estimated with distribution-based methods has several disadvantages. It overlooks clinical significance and depends on the sample variability, so it fails to fulfill the original intention of the MCID, which to distinguish clinical from statistical significance. Moreover, as yet there is no consensus on which method provides a better estimation of MCID. In this study, we have employed several complementary distribution-based methods, and the results are presented as preliminary estimates of MCID. Finally, the range and weighted mean value of the MCID were estimated using the anchor-based method. The weight used here was the correlation coefficient between the EQ-5D utility index and each anchor.

## Results

### Descriptive data of the sample

A total of 298 patients completed the study. Table 1 summarizes the clinical and demographic characteristics of patients according to the GOLD severity classification. Findings show that 32 patients had mild COPD; 156 patients, who formed the majority, moderate COPD; 90 patients severe COPD; and 20 patients very severe COPD. The mean age was about 69 years, and 86% of the participants were male. The mean BMI was 22.8 kg/m^2^, and the higher the severity of COPD, the lower the BMI, with significant differences among groups. Further, 26% of all patients and 75% of GOLD 4 patients had been treated with three or more therapies. The mean frequency of acute exacerbations over the past year was 0.5, and 15.8% of all patients received hospital treatment more than twice a year, or more than one admission a year, due to acute exacerbation. With reference to comorbidities, 47% of the patients had cardiovascular disease and 37% had hypertension. None of the comorbidities showed a difference in the quality of life score of the patients based on severity groups.

**Table 1:** Characteristics of the study participants

	All	GOLD 1 mild	GOLD 2 moderate	GOLD 3 severe	GOLD 4 very severe
	(*N* = 298)	(*N* = 32)	(*N* = 156)	(*N* = 90)	(*N* = 20)
Parameter	Mean, N (SD, %)				
Age (yr)	69.2 ± 8.8	70.9 ± 8.2	69.5 ± 8.7	68.8 ± 8.8	65.5 ± 10.1
Male (n,%)	257 (86.2%)	31 (96.9%)	129 (82.7%)	80 (88.9%)	17 (85.0%)
BMI (kg/m2) *	22.8 ± 3.3	24.0 ± 3.1	23.4 ± 3.1	22.1 ± 3.3	20.1 ± 3.2
Medical aid (n,%)	27 (9.1%)	1 (3.1%)	15 (9.6%)	7 (7.8%)	4 (20.0%)
Unemployed (n,%)	240 (80.5%)	24 (75.0%)	129 (82.7%)	71 (78.9%)	16 (80.0%)
Smoking history (pack-yr)	43.0 ± 28.7	42.0 ± 53.0	41.1 ± 24.7	47.0 ± 23.1	40.5 ± 18.9
Time from diagnosis (yr) *	5.4 ± 3.7	3.5 ± 2.5	4.8 ± 3.3	6.4 ± 4.0	8.7 ± 4.4
History of Tuberculosis (n, %) *	103 (34.6%)	6 (18.8%)	49 (31.4%)	35 (38.9%)	13 (65.0%)
Treatment					
LABA+LAMA (n, %) *	66 (22.1%)	4 (12.5%)	29 (18.6%)	25 (27.8%)	8 (40.0%)
ICS/LABA (n, %) *	114 (38.3%)	6 (18.8%)	46 (29.5%)	51 (56.7%)	11 (55.0%)
Treated with 3 or more therapies (n, %) *	77 (25.8%)	0 (0.0%)	27 (17.3%)	35 (38.9%)	15 (75.0%)
Exacerbation					
≥ 2 for exacerbations in past year *	29 (9.7%)	2 (6.2%)	7 (4.5%)	14 (15.6%)	6 (30.0%)
admission ≥2 or hospitalized≥1 for exacerbations in past year *	47 (15.8%)	3 (9.4%)	14 (9.0%)	21 (23.3%)	9 (45.0%)
History of Exacerbations (n-yr) *	0.5 ± 1.3	0.3 ± 0.6	0.3 ± 1.2	0.6 ± 1.3	1.4 ± 2.3
Post-bronchodilater lung function					
FEV_1_ (ml) *	1.6 ± 0.6	2.5 ± 0.4	1.8 ± 0.4	1.2 ± 0.3	0.7 ± 0.2
FEV_1_ (% predicted) *	55.8 ± 17.9	88.1 ± 8.3	62.2 ± 8.1	40.1 ± 5.5	25.4 ± 3.5
FVC (ml) *	3.0 ± 0.8	3.6 ± 0.7	3.1 ± 0.8	2.7 ± 0.7	2.3 ± 0.8
FEV_1_/FVC (%) *	54.3 ± 15.1	68.9 ± 7.7	59.1 ± 12.0	45.0 ± 13.6	34.9 ± 7.8
Comorbidities treated in hospital (n,%)					
CVD (Angina, MI, CHF, AF)	140 (47.0%)	17 (53.1%)	75 (48.1%)	40 (44.4%)	8 (40.0%)
Hypertension	110 (36.9%)	15 (46.9%)	63 (40.4%)	29 (32.2%)	3 (15.0%)
Diabetes	44 (14.8%)	5 (15.6%)	22 (14.1%)	13 (14.4%)	4 (20.0%)
GERD	75 (25.2%)	9 (28.1%)	42 (26.9%)	21 (23.3%)	3 (15.0%)
Anxiety / depression	20 (6.7%)	1 (3.1%)	7 (4.5%)	9 (10.0%)	3 (15.0%)
Asthma	67 (22.5%)	6 (18.8%)	39 (25.0%)	17 (18.9%)	5 (25.0%)
Number of comorbidities (n)	1.6 ± 1.5	1.7 ± 1.6	1.7 ± 1.5	1.5 ± 1.4	1.2 ± 1.2

SD: standard deviation; BMI: body mass index; Medical aid: tax-based health security system which targets low income group; LABA: long acting bronchodilator; LAMA: long acting muscarinic antagonist; ICS: inhaled corticosteroid; FEV_1_: forced expiratory volume in 1 s: FVC: forced vital capacity; CVD: cardiovascular disease; MI: myocardial infarction; CHF: congestive heart failure; AF: atrial fibrillation; GERD: gastroesophageal reflux disease;

* *p* < 0.05, *p*-value was calculated to test significant differences between severity groups using Chi-square test for categorical variables and ANOVA for continuous variables

### Construct validity

The EQ-5D utility index score, EQ-VAS, and CAT scores are presented as mean scores according to the GOLD severity group (Table 2). All the quality of life scores decreased with an increase in disease severity, and there were a statistically significant differences between groups. The post-hoc analysis revealed that the scores on the EQ-5D utility index and CAT differed between all neighboring groups, except between GOLD 1 and GOLD 2. The effect size was computed to identify the degree of difference in the quality of life of patients by severity group. Standardized mean differences between neighboring groups were greater than 0.2, which confirmed that the severity of pulmonary function affects the quality of life of patients. In particular, even between the GOLD 1 and GOLD 2 groups, which did not reveal a statistically significant difference in quality of life scores, there were moderate differences in the EQ-5D utility index according to the effect size analysis. Among the quality of life instruments used in this study, the EQ-5D utility index best captured the differences in quality of life between the GOLD 1 and GOLD 2 groups and between the GOLD 3 and GOLD 4 groups. On the other hand, the CAT score was best at discriminating between the GOLD 2 and GOLD 3 groups. Additionally, among the quality of life instruments, the EQ-5D utility index was most highly correlated with lung function (Spearman’s ρ = 0.422 and − 0.380 for the FEV_1_% predicted and the GOLD severity grade, respectively; Pearson’s correlation coefficients are similar to Spearman’s coefficients).

**Table 2:** HRQoL scores and effect size according to the GOLD severity

	EQ-5D utility* Mean score (SD)	EQ-VAS* Mean score (SD)	CAT* Mean score (SD)
GOLD classification of lung function and HRQoL			
GOLD-1, FEV_1_ ≥ 80% predicted	0.911^a^ (0.111)	72.97^a^ (11.49)	12.28^a^ (7.42)
GOLD-2, 50% ≤ FEV_1_ < 80% predicted	0.860^a^ (0.117)	68.84^ab^ (17.18)	14.14^a^ (8.26)
GOLD-3, 30% ≤ FEV_1_ < 50% predicted	0.792^b^ (0.156)	63.44^b^ (19.2)	19.76^b^ (8.62)
GOLD-4, FEV_1_ < 30% predicted	0.664^c^ (0.169)	52.50^c^ (16.5)	24.85^c^ (7.78)
Total	0.831 (0.146)	66.52 (17.85)	16.39 (8.96)
Effect size ^d^			
Between GOLD-1 and GOLD-2	0.445	0.252	−0.228
Between GOLD-2 and GOLD-3	0.510	0.301	−0.670
Between GOLD-3 and GOLD-4	0.803	0.584	−0.601
Correlations for each HRQoL scores ^e^			
Between FEV_1_% predicted	0.422	0.284	−0.371
Between GOLD grade	−0.380	−0.258	0.375

*HRQoL* Health-Related Quality of Life, *SD* standard deviation, *FEV*_*1*_ forced expiratory volume in 1 s

*p < 0.001, p-value for severity group was calculated by ANOVA;

^a; b; c;^ the same superscript letters indicate non-significant difference between GOLD groups based on Duncan’s post-hoc test (p < 0.05);

^d^effect size used Cohen’s d = M_1_ - M_2_ / S_pooled_, where S_pooled_ = √[(S_12_+ S_22_) / 2];

^e^Spearman’s rank correlation coefficient (*p* < 0.05)

Table 3 presents the mean quality of life score by GOLD group after controlling for factors that may affect the quality of life apart from COPD severity. When controlling for age, gender, number of pack-years of smoking, insurance type, employment status, and number of comorbidities, the differences in the quality of life scores between groups decreased compared to those computed without controlling for these factors. However, statistically significant differences remained. Post-hoc analysis of the differences between neighboring groups showed similar results as in the former analysis. However, CAT scores did not differ significantly between the GOLD 3 and GOLD 4 groups after adjustment.

**Table 3:** HRQoL scores after controlling factors affecting the quality of life of patients with COPD

	EQ-5D utility*	EQ-VAS*	CAT*
	Mean score (95% confidence interval)		
GOLD-1, FEV_1_ ≥ 80% predicted	0.900 ^a^ (0.855, 0.944)	73.38 ^a^ (67.15, 79.62)	12.86 ^a^ (9.96, 15.77)
GOLD-2, 50% ≤ FEV_1_ < 80% predicted	0.857 ^a^ (0.836, 0.879)	67.73 ^ab^ (64.74, 70.72)	13.95 ^a^ (12.55, 15.34)
GOLD-3, 30% ≤ FEV_1_ < 50% predicted	0.793 ^b^ (0.766, 0.821)	63.32 ^b^ (59.48, 67.16)	19.75 ^b^ (17.96, 21.53)
GOLD-4, FEV_1_ < 30% predicted	0.664 ^c^ (0.601, 0.728)	51.08 ^c^ (42.25, 59.91)	24.08 ^b^ (19.97, 28.19)

*HRQoL* Health-Related Quality of Life, *FEV*_*1*_ forced expiratory volume in 1 s:

*p < 0.001, *p*-value for severity group was calculated by ANCOVA; Covariates: age, gender, smoke years, insurance type, employment status, number of comorbidities;

^a; b; c;^ the same superscript letters indicate non-significant difference between GOLD groups based on LSD post-hoc test (p < 0.05)

### MCID

The preliminary estimates of MCID using the distribution-based method was 0.073, 0.091, and 0.029 for the 0.5*SD, SEM, and Cohen’s approach, respectively. A simple linear regression was used to estimate the MCID by clinically relevant external criteria (anchor) (Table 4). The coefficient corresponding to the difference between 2 and 3, which is known as the MCID of the CAT score, was 0.021–0.031 (95% confidence interval (CI): 0.018–0.035). When the EQ-VAS score or FEV_1_% predicted value was used as an external criterion, the respective estimates were 0.033 (95% CI: 0.027–0.040) and 0.017–0.033 (95% CI: 0.012–0.042). The external criterion most relevant to the utility score was the CAT score (adjusted *R*^2^ = 0.41). The MCID range for the participants’ EQ-5D utility index scores was 0.017–0.033 (95% CI: 0.012–0.042), and the pooled estimation using the correlation coefficient was 0.028 (95% CI: 0.023–0.034). This was consistent with the MCID estimated by the Cohen’s effect size distribution method. Even after adjusting for other factors affecting quality of life (Table 3), we confirmed that all the utility differences between groups exceeded the minimal clinically importance difference. In contrast, for the CAT and EQ-VAS scores, not all the differences between groups exceeded the MCID.

**Table 4:** MCID estimates for the EQ-5D utility index and anchor

	Estimated value for MCID		
Anchor	MCID	(95% CI)	Correlation coefficient^a^
2–3 CAT total score changes	0.021–0.031	(0.018–0.035)	0.651
8 EQ-VAS score changes	0.033	(0.027–0.040)	0.581
5–10% FEV_1_ predicted changes	0.017–0.033	(0.012–0.042)	0.422
The pooled MCID estimate^b^	0.028	(0.023–0.034)	NA

*MCID* minimal clinically important differences, *CI* confidence interval, *FEV*_*1*_ forced expiratory volume in 1 s

^a^The relevance of each anchor to the EQ-5D utility index was computed as Spearman’s rank correlation coefficient

^b^the pooled estimation was calculated as weighted mean using the correlation coefficient between the EQ-5D utility index and each anchor

## Discussion

In this study, the health-related quality of life of patients with COPD was measured using the EQ-5D utility index, EQ-VAS, and CAT. Among these instruments, we examined whether the EQ-5D utility index is able as a general instrument to discriminate the GOLD severity groups. We estimated the minimal clinically important difference in the EQ-5D utility index scores, which would be a meaningful for patients with COPD.

We found that the EQ-5D utility, EQ-VAS, and CAT scores differed significantly by COPD severity, and this differentiation was obvious even when controlling for confounding variables. In addition to the tests of variance and covariance, this result was also consistent with the findings of the analysis of the effect size using Cohen’s *d*. In particular, the performance of the EQ-5D utility index on assessing and differentiating quality of life was not inferior to that of the CAT, which is a COPD-specific instrument. Further, the correlation of the EQ-5D utility index with pulmonary function was higher than that of the other two measures. Preference-based utility valuation may not consider the impact of medical conditions on individual patients, and previous studies have reported that MAU measures such as the EQ-5D utility index have limitations in detecting HRQoL changes or differences in the COPD population [4, 13, 14]. Especially in the EQ-5D-3L descriptive system, ceiling effects have been observed due to the limitation of three response categories per item and lack of important health-related quality of life dimensions such as vitality [42, 43]. In the present study, the failure of the EQ-5D utility index to statistically differentiate between the mild and moderate COPD groups could be attributed to this ceiling effect, which would have affected the reporting of the relatively mild health status of patients. In this study, 50% (16/32) of the participants in the mild group and 31.4% (49/156) of those in the moderate group reported full health (11111). Indeed, the ceiling effect is a limitation of the EQ-5D utility index that may be somewhat improved in the 5 L system [44]. However, since a valuation study for EQ-5D-5 L in South Korea had not been completed at the time of the present survey, it was not used.

Despite the instrumental constraints of the EQ-5D-3L, our results suggest that generic MAU measures are as discriminable as condition-specific measures. The CAT was developed to replace the Saint George’s Respiratory Questionnaire (SGRQ), which is a complex and time-consuming tool for evaluating the quality of life of patients with COPD. However, the SGRQ has also been reported to show weak correlations with physiological indices such as FEV_1_ [45]. While the CAT and SGRQ mainly deal with physical functioning and symptoms, MAU measures, such as the EQ-5D utility index, include social or mental functioning such as usual activity, anxiety, and depression. Therefore, they seem to reflect the effect of lung function impairment on the patient’s health-related quality of life more comprehensively. However, this result should not be extended to the individual patient level.

Previous studies have reported conflicting results for differences in the quality of life scores of moderate and severe COPD groups. Some authors have noted that this is because a trial population tends to show less variation than a real-life population does owing to application of a strict protocol [46]. The present study was designed to investigate the quality of life of patients with COPD. Although the patients included in this study were not a population-based sample representative of the true population distribution, the inclusion/exclusion criteria were not strictly limited. Therefore, we could include a variety of patients. Additionally, only the patients with a stable status were selected, and we also ensured that at least 20 patients were recruited per GOLD category. Therefore, we were able to obtain meaningful results in terms of differentiation that were significant even after adjusting for other factors. However, this study could also be confounded by limitations pertaining to the hospital setting, such as too few cases of mild patients. Therefore, we could not confirm the significance of the difference in health-related quality of life between the mild and moderate COPD groups. However, we did find a moderate difference between the two groups in terms of effect size, which is relatively independent of the sample size. In South Korea, the use of tertiary hospitals, even in the regular management of chronic diseases, is common. Overall, the present study reported higher utility values than those reported in previous studies, and there might be differences in the applied valuation methods or national algorithms. A meta-analysis study acknowledged a considerable amount of variation across studies. However, there was consensus on the finding that the greater the severity, the greater the degree of deterioration of quality of life [10, 11, 47].

Interest in the MCID of the EQ-5D utility index has increased in recent years, but there is no widely accepted value or range. Further, the MCID of the EQ-5D utility index score of patients with COPD was included as a subgroup in the study conducted by Walters and Brazier [48], but it did not yield a significant result by itself. Therefore, the present study is meaningful in that it identified the minimally important difference in the quality of life of patients with COPD as measured by the EQ-5D utility index. This empirical evidence could be used in the process of establishing the MCID for the EQ-5D utility index. Furthermore, it is recommended that the estimation of the MCID should be based on multiple approaches or on the triangulation of methods rather than using one approach alone [28, 49]. In the present study, we obtained a pooled estimate of the MCID of the EQ-5D utility index of 0.028 (range: 0.017–0.033) for patients with COPD using several relevant patient-rated and disease-specific variables as anchors. This finding was supported through the distribution-based method that employed Cohen’s approach. In a study of the MAUT instrument with patients with rheumatoid arthritis, the MID estimates obtained using anchor-based methods were also consistent with those determined by the effect size method [50]. Furthermore, some studies reported an MCID of 0.03 for the EQ-5D utility index score of patients with COPD, which is similar to that observed in the present study [4, 14, 51]. Coretti et al. [29] found a remarkable heterogeneity in the methods of estimation of the MCID for the EQ-5D utility index and acknowledged that they may vary according to population and context. Thus, further discussion and research is needed to reach a consensus on the MCID for the EQ-5D utility index.

## Conclusions

The present study examined the quality of life of patients with COPD using data obtained from a cross-sectional patient survey. Additionally, it examined the validity of the EQ-5D utility index both statistically and clinically. In conclusion, the EQ-5D utility index, a general instrument, exhibited a good ability to distinguish between patients based on COPD severity with a performance similar to that of the CAT, a COPD-specific instrument. The MCID estimates for the EQ-5D utility index using both distribution and anchor-based methods were similar. The pooled MCID estimate for the EQ-5D utility index was 0.028. Given that the differences across all groups exceeded the MCID, the EQ-5D utility index also seems to be capable of capturing significant clinical differences. Finally, the utility score measured by the EQ-5D seems appropriate for use in the economic evaluation of patients with COPD.

## Supplementary information

**Additional file 1: S1**. HRQoL median score according to the GOLD severity group.

## Data Availability

The data that support the findings of this study are available from corresponding author.

## Abbreviations

HRQoL: Health-Related Quality of Life
COPD: Chronic Obstructive Pulmonary Disease
GOLD: Global Initiative for Chronic Obstructive Lung Disease
MAU(T): Multi-attribute utility (theory)
CSM: Condition-specific measure
MCID: Minimal clinically important differences
PROs: Patient reported outcomes
IRB: Institutional Review Board
FEV_1_: Forced expiratory volume in 1 s
FVC: Forced vital capacity
BMI: Body mass index
LABA: Long-acting β2-agonists
LAMA: Long-acting muscarinic antagonist
ICS: Inhaled corticosteroid
CVD: Cardiovascular disease
MI: Myocardial infarction
CHF: Congestive heart failure
AF: Atrial fibrillation
GERD: Gastroesophageal reflux disease
CI: Confidence interval
SD: Standard deviation
SE: Standard error
SEM: Standard error of measurement
ICC: Intraclass correlation coefficient
ANOVA: Analysis of variance
ANCOVA: Analysis of covariance
LSD: Least significant difference
EQ-5D-3L: Three-level five-dimensional European Quality of Life (EuroQol) scale
VAS: Visual Analog Scale
CAT: Chronic Obstructive Pulmonary Disease Assessment Test
TTO: Time trade-off
mMRC: Modified Medical Research Council
SGRQ: Saint George’s Respiratory Questionnaire
