# Supplementary material for: Validity of EQ-5D utility index and minimal clinically important difference estimation among patients with chronic obstructive pulmonary disease
Source: BMC Pulm Med. 2020 Mar 23;20:73. doi: 10.1186/s12890-020-1116-z (PMC7092534; doi:10.1186/s12890-020-1116-z)
Supplement: Supplementary file 1 — Additional file 1: S1. HRQoL median score according to the GOLD severity group. [file 12890_2020_1116_MOESM1_ESM.docx]

|  | EQ-5D utility* | EQ-VAS* | CAT* |
| --- | --- | --- | --- |
|  | Median score  (IQR) | | |
| GOLD-1, FEV1 ≥ 80% predicted | 0.957 **^a^**  [0.839; 1] | 70 **^a^**  [70; 80] | 9.5 **^a^**  [7; 18] |
| GOLD-2, 50% ≤FEV1 < 80% predicted | 0.854 **^b^**  [0.760; 1] | 70 **^a^**  [60; 80] | 14 **^a^**  [7; 19.5] |
| GOLD-3, 30% ≤FEV1 < 50% predicted | 0.803 **^c^**  [0.720; 0.904] | 60 **^b^**  [50; 80] | 20.5 **^b^**  [14; 26] |
| GOLD-4, FEV1 < 30% predicted | 0.714 **^d^**  [0.677; 0.759] | 50 **^c^**  [40; 55] | 25.5 **^c^**  [18.5; 30] |

S1. HRQoL median score according to the GOLD severity group

IQR: Inter Quartile Range [Q1; Q3];

*p<0.001, p Value for severity group was calculated by Kruskal-Wallis test;

a; b; c; the same superscript letters indicate non-significant difference between GOLD groups based on Wilcoxon rank-sum test (p<0.05)
